# SKA3 promotes cell proliferation and migration in cervical cancer by activating the PI3K/Akt signaling pathway

**DOI:** 10.1186/s12935-018-0670-4

**Published:** 2018-11-14

**Authors:** Rong Hu, Ming-qing Wang, Wen-bo Niu, Yan-jing Wang, Yang-yang Liu, Ling-yu Liu, Ming Wang, Juan Zhong, Hai-yan You, Xiao-hui Wu, Ning Deng, Lu Lu, Lian-bo Wei

**Affiliations:** 1 Shenzhen Hospital, Southern Medical University, No. 1333, Xinhu Road, Bao’an District, Shenzhen, 518101 Guangdong China; 2 School of Traditional Chinese Medicine, Southern Medical University, No. 1838, Guangzhou Avenue North, Baiyun District, Guangzhou, 510515 Guangdong China; 3 Zhujiang Hospital of Southern Medical University, No. 253, Industrial Avenue, Haizhu District, Guangzhou, 510280 Guangdong China; 4 The First Affiliated Hospital, Guangzhou University of Chinese Medicine, No.16 Baiyun Airport Road, Baiyun District, Guangzhou, 510405 Guangdong China; 5 Cancer Research Institute, Southern Medical University, No. 1838, Guangzhou Avenue North, Baiyun District, Guangzhou, 510515 Guangdong China; 6 Zhongshan Huangpu People’s Hospital, No. 32, Long’an Street, Huangpu Town, Zhongshan, 528429 Guangdong China

**Keywords:** SKA3, Cervical cancer, Cell proliferation, Cell cycle, PI3K/Akt

## Abstract

**Background:**

Cervical cancer (CC) is one of the most common cancers among females worldwide. Spindle and kinetochore-associated complex subunit 3 (SKA3), located on chromosome 13q, was identified as a novel gene involved in promoting malignant transformation in cancers. However, the function and underlying mechanisms of SKA3 in CC remain unknown. Using the Oncomine database, we found that expression of SKA3 mRNA is higher in CC tissues than in normal tissues and is linked with poor prognosis.

**Methods:**

In our study, immunohistochemistry showed increased expression of SKA3 in CC tissues. The effect of SKA3 on cell proliferation and migration was evaluated by CCK8, clone formation, Transwell and wound-healing assays in HeLa and SiHa cells with stable SKA3 overexpression and knockdown. In addition, we established a xenograft tumor model in vivo.

**Results:**

SKA3 overexpression promoted cell proliferation and migration and accelerated tumor growth. We further identified that SKA3 is involved in regulating cell cycle progression and the PI3K/Akt signaling pathway via RNA-sequencing (RNA-Seq) and gene set enrichment analyses. Western blotting results revealed that SKA3 overexpression increased levels of p-Akt, cyclin E2, CDK2, cyclin D1, CDK4, E2F1 and p-Rb in HeLa cells. Additionally, the use of an Akt inhibitor (GSK690693) significantly reversed the cell proliferation capacity induced by SKA3 overexpression in HeLa cells.

**Conclusions:**

We suggest that SKA3 overexpression contributes to CC cell growth and migration by promoting cell cycle progression and activating the PI3K–Akt signaling pathway, which may provide potential novel therapeutic targets for CC treatment.
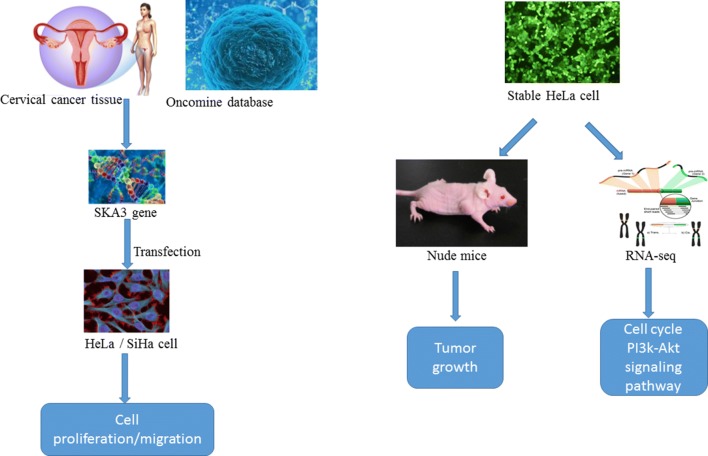

**Electronic supplementary material:**

The online version of this article (10.1186/s12935-018-0670-4) contains supplementary material, which is available to authorized users.

## Background

Cervical cancer (CC) is the second most common type of gynecologic cancer worldwide [1], with approximately 500,000 newly diagnosed cases and 275,000 deaths every year [2]. Depending on the stage of the disease, 5-year survival rate ranges from approximately 5–50%, depending on the stage [3]. Furthermore, due to poor economic situations and delays in treatment, morbidity and mortality rates of CC remain very high in some developing countries due to poor economic situations and delays in treatment [4, 5]. It is well known that persistent infection with HPV is a major risk factor for CC due to the oncoproteins E6 and E7. These factors inactivate and degrade tumor suppressor p53 and retinoblastoma (Rb), causing cell cycle deregulation, genomic instability, and increased chromosomal aberrations and mutations in cellular genes [6]. Gene network reconstruction has revealed cell cycle and antiviral genes as major drivers of CC [7]. Current standard treatments for CC, including surgery and definitive chemoradiation, result in the loss of childbearing ability [8], and targeted therapeutic strategies have mainly focused on the HPV E6 and E7 oncogenic proteins [9]. Nevertheless, the outcome of current therapy strategies is still poor. Therefore, investigating the exact molecular mechanisms of CC may promote the identification of novel biomarkers and treatment targets, which is critical for improving the prognosis of these patients [10].

SKA3, a subunit located in the kinetochore outer layer of the SKA complex, is not only required for controlling and promoting proper mitotic exit during mitosis by cooperating with the NDC80 complex [11, 12] but also plays an important role in meiotic spindle migration and anaphase spindle stability [13]. Previous studies have reported that SKA3 participates in cancer pathogenesis and progression. SKA3 is frequently somatically mutated in breast cancer and has a role in cell growth [14]. A recent study showed that SKA3 is associated with patient outcome and aggressive disease development in several cancers [15]. By analyzing an Oncomine dataset, we found that SKA3 mRNA expression is higher in CC tissue than in normal tissue and may be associated with survival rate in CC patients. However, the detailed functions and underlying mechanisms of SKA3 in CC remain largely unknown.

Cell cycle progression critically depends on numerous regulatory processes that are often dysregulated in cancer [16]. Cyclin D in complexes with CDK4 or CDK6 and cyclin E in a complex with CDK2 regulate progression through the G1-S boundary of the cell cycle. These complexes phosphorylate and thereby prevent Rb from binding to E2F, which once released, drives cells from G1 into S phase [17, 18].

Some signaling pathways have been found to have important functions in the occurrence and progression of CC, such as the Notch1 ligand, Wnt/beta-catenin, p53, p38 MAPK, and PI3K/Akt/mTOR signaling pathways [19–22]. Overall, a deeper knowledge of signal transduction may provide new targets for tumor therapy. The phosphoinositide 3-kinase (PI3K)/Akt pathway is a classical and important signaling pathway involved in numerous cellular functions, including cell proliferation, survival, adhesion, migration and metabolism [23, 24]. Furthermore, PI3K/Akt signaling pathway controls proliferation, transformation, growth, apoptosis, drug resistance, and other processes in various types of cancers [25, 26]. Previous studies have shown that PI3K/Akt signaling pathway is closely associated with the occurrence and development of CC [27], and the pathway has become a potential target for the prevention and treatment of CC [28, 29].

This study was designed to investigate the role and mechanisms of SKA3 in CC both in vitro and in vivo. We hypothesized that SKA3 may play a very important role in the development and progression of CC. Importantly, SKA3 expression may indicate a poor prognosis and could serve as a potential therapeutic target in CC.

## Materials and methods

### Cell culture

HEK 293FT cells and human CC cell lines (HeLa, C4-I, CaSki, C-33A, HT-3, SiHa, SW756, MS751, ME-180) were purchased from the Chinese Academy of Science cell bank (Shanghai, China). The cell lines were authenticated by a Cell Line Authentication Service with an STR Profile Report (Genetic Testing Bio-technology, Suzhou, China). All CC cell lines were cultivated in RPMI-1640 medium supplemented with 10% fetal bovine serum (FBS) (Gibco, BRL), 100 units/mL penicillin and 100 μg/mL streptomycin (Gibco, New York, USA). HEK 293FT cells were maintained in DMEM (Gibco, BRL) supplemented with 10% FBS in a humidified atmosphere containing 5% CO_2_ at 37 °C.

### Real-time quantitative PCR (RT-qPCR)

Total RNA was extracted using TRIzol reagent (TaKaRa, Dalian, China) and reverse transcribed into cDNA using a Prime Script RT Reagent Kit (TaKaRa), according to the manufacturer’s instructions. RT-qPCR was performed with a CFX96™ Real-Time System (Bio-Rad Hercules, California, USA) using SYBR Green (SYBR Premix Ex Taq™ II; TaKaRa) for fluorescent quantification. The following cycling conditions were used: pre-denaturation at 95 °C for 30 s, 35 cycles of denaturation at 95 °C for 5 s, 35 cycles), annealing at 55–60 °C for 30 s, extension at 72 °C for 1 min) and a final extension at 72 °C for 10 min. Relative mRNA expression was calculated using the 2^−∆∆Ct^ method. The primers of SKA3 and β-actin were list in Table 1.

**Table 1:** Sequences of primers and short RNA oligos

Gene	Forward primers	Reverse primers
SKA3	5′-CAGATCCCTCTTCACCTACGA-3′	5′-TCAACGTTTAAAGGGGGACA-3′
β-Actin	5′-GGCATCCTCACCCTGAAGTA-3′	5′-GGGGTGTTGAAGGTCTCAAA-3′
Over-SKA3	5′-GCTCTAGAGCCACCATGGACCCTATCCGGAGCTTCTGC-3′	5′-CGGAATTCTCACTTGTCATCGTCATCCTTGTAGTCGTTTTCTTTGTTGCTGACATCTCGGATG-3′
Sh-SKA3	CCGGCATGGACAGAACATCCGAGATCTCGAGATCTCGGATGTTCTGTCCATGTTTTTTG	

### Clinical specimens

A tissue microarray containing 100 samples of formalin-fixed, paraffin-embedded (FFPE) CC and para-carcinoma tissues with detailed clinical characteristics was purchased from Alenabio.com (CR1003, Xi an, China). Forty CC tissues and 10 normal tissues (each tissue was represented twice) were included on the tissue chip, which was evaluated by IHC staining to examine SKA3 expression. The 7th edition of the American Joint Committee on Cancer (AJCC) Cancer Staging Manual was used to reclassify the tumor-node-metastasis (TNM) staging. The clinical features of all patients are listed in Table 2. All procedures involving human subjects were performed in accordance with institutional and national research committee ethical standards.

**Table 2:** Clinical and pathological variables and the expression of SKA3 in 40 cases of cervical cancer

Parameters	Expression of SKA3		p-value
	Low	High	
Age (years)			0.3598
≥ 50	2 (5%)	15 (37.5%)	
< 50	4 (10%)	19 (47.5%)	
TNM stage			0.999
I + II	6 (15%)	32 (80%)	
III + IV	0 (0%)	2 (5%)	
Clinical stage			0.999
I + II	5 (12.5%)	29 (72.5%)	
III + IV	1 (2.5%)	5 (12.5%)	

### Overexpression plasmid construction and lentivirus infection

The coding sequence of the SKA3 gene was obtained from PUBGENE. The primers were obtained from The Beijing Genomics Institute (BGI) and were listed in Table 1. The SKA3 gene was first cloned into the PCDH lentiviral vector. After enzyme digestion and DNA sequencing, a recombinant plasmid containing a GFP reporter gene and FLAG tag was successfully constructed. The packaging plasmids pMD2.G and pSPAX2 were mixed with PCDH-SKA3 or PCDH-NC (control). All plasmids were transfected into HEK 293FT cells using Lipofectamine 2000 reagent (Invitrogen) to form lentiviral particles and generate stably transfected cell lines. Lentiviral particles were harvested and used to infect HeLa cells 48 h later. After selection with puromycin (1 mg/mL) (Sigma-Aldrich) for 2 weeks, western blotting was performed to validate SKA3 overexpression or knockdown in the stably transfected cells. SKA3 knockdown lentiviral particles were purchased from Genechem, and the sequence was listed in Table 1.

### CCK8 assay and clone formation assay

For the Cell Counting Kit-8 assay (CK04, Dojindo Kumamoto, Japan), stable HeLa cell lines were seeded at 5000 cells per well in 96-well plates. Cell viability was measured at 450 nm using a spectrophotometric plate reader from 0 to 7 days, and each experiment was performed in triplicate. For the clone formation assay, cells were plated at 500 cells per well in 6-well plates and cultured for 14 days. Clones were counted under an inverted microscope after the cells were fixed in methyl alcohol and stained with 0.5% crystal violet (Saiguo, Guangzhou, China). Clones containing > 50 cells were counted for statistical analysis. Cells in each group were plated in three duplicate wells, and all experiments were repeated independently three times.

### Wound healing assay

Stable HeLa cells were plated in 6-well cell culture plates (3 × 10^6^ cells per well) and grown to near 100% confluence. The monolayers were scratched with a sterile 200-μL tip and washed with phosphate-buffered saline (PBS) to remove the detached cells. Then, the cells were then cultured in serum-free medium for 48 h. Images of cells migrating at the corresponding wound sites were captured at 0, 24, and 48 h using an inverted microscope (200×), and the wound size was measured by Image-pro plus 6.0. Data were collected from three independent experiments.

### Transwell migration assays

The transwell migration assay was carried out using 24-well MILLI cell Hanging Cell Culture Inserts (8 μm) (Corning, Bedford, MA, USA). The upper surface of Transwell chambers (8-mm pores; Corning) was used to assess the cell migration ability. Dishes were placed in a cell culture incubator for 1 h at 37 °C. Stable HeLa cells were harvested in 200 µL serum-free medium and plated in the upper chamber at a density of 1 × 10^5^ cells per insert. The lower chambers were filled with 600 µL of medium supplemented with 20% FBS. After 24 h of incubation, the migrated cells on the membrane surface were fixed with methanol, stained with 0.5% crystal violet, and counted under an inverted microscope (200×). Data were collected from three independent experiments.

### Flow cytometry to detect the cell cycle and apoptosis

Stable HeLa cells (3 × 10^5^ cells per well) were plated in 6-well cell culture plates. For cell cycle analysis, cells were collected by trypsinization after 2 days, washed once with PBS, fixed in 70% alcohol at 4 °C overnight, and again washed with PBS. RNA helicase was added to the cells for 30 min at 37 °C. The cells were then stained with 400 µL of propidium iodide (PI) buffer (Chemo Metec, Allerod, Denmark), kept in a darkroom at 4 °C for 30 min, and evaluated using a FACS flow cytometer (BD, Franklin Lakes, NJ, USA). For cell apoptosis analysis, cells were collected after transfection by trypsinization (without EDTA), washed once with PBS, added to 500 µL of 1× binding buffer, and stained with 5 µL of Annexin V-PE and 5 µL of 7-AAD. The mixture was incubated in the dark for 15 min and detected by flow cytometry. All experiments were repeated independently three times.

### In vivo xenograft tumor model

BALB/c nude mice (4–6 weeks old, male) were purchased from the Experimental Animal Centre of Southern Medical University and maintained under standard pathogen-free conditions, with 6 mice in each group. A sample of 1 × 10^7^ HeLa cells with stable SKA3 overexpression or SKA3 knockdown or control plasmid re-suspended in 200 µL of PBS was injected into the left, middle or right dorsal flank of the mice, respectively. To analyze tumor growth, tumor size was measured using calipers for 6 weeks according to the following formula: L*W*W*π/6, where L is the length and W is the width of the tumor. The tumor tissues were harvested, fixed, and paraffin-embedded before 4-mm tissue sections were obtained. All sections were subjected to immunohistochemistry (IHC) staining. All animal studies (including the mouse euthanasia procedure) were performed in compliance with the regulations and guidelines of Southern Medical University Institutional Animal Care and according to AAALAC and IACUC guidelines.

### Immunohistochemistry (IHC) staining

IHC staining was performed on formalin-fixed paraffin-embedded tumor tissue sections and a clinical tissue microarray chip. The sections were deparaffinized and rehydrated, and endogenous peroxidase activity was blocked by incubating the samples with 3% H_2_O_2_ for 15 min in the dark. Antigen retrieval was performed by heating in a pressure cooker in citrate buffer (Saiguo, Guangzhou, China) for 10 min. The samples were then incubated at room temperature for 40 min and washed three times with PBS. Next, 5% BSA was used to block nonspecific binding at room temperature for 30 min. The processed sections were incubated with a primary antibody overnight at 4 °C at the following dilutions: SKA3, 1:800 for the clinical tissue microarray chip and 1:200 for tumor sections (Abcam, Cambridge, MA, USA); Ki67, 1:100 (Abcam, Cambridge, MA, USA); Cyclin D1, 1:50 (Abcam, Cambridge, MA, USA); and CDK4, 1:50 (Abcam, Cambridge, MA, USA). The sections were washed 3 times with PBS and incubated with a biotinylated secondary antibody for 40 min at room temperature. Immunostaining signals were enhanced and visualized using the ABC staining system and DAB substrate kit (Vector Laboratories, CA, USA) according to the manufacturer’s instructions. Signal intensity was scored as follows: 0 (no staining), 1 (weak staining), 2 (moderate staining), and 3 (strong staining). The percentage of positively stained cells was divided into four categories: < 25% (1), 25–50% (2), 51–75% (3), and > 76% (4). The final staining scores were calculated as the intensity × the staining percentage to achieve a score between 0 and 12. A final score > 6 was defined as high expression, and ≤ 6 was defined as low SKA3 expression.

### RNA-sequencing and gene set enrichment analysis (GSEA)

Total mRNA was extracted from HeLa cells stably overexpressing SKA3 or PCDH using an RNA extraction kit (Qiagen). RNA-Seq was performed using an Ion Proton system for next-generation sequencing according to the manufacturer’s instructions. Sequenced reads were mapped to the hg19 genome using the Ion Torrent TMAP aligner with the ‘map4’ option. HTSeq-Count was used to quantify the aligned RNA-Seq reads against exon regions of genes in the RefSeq hg19 annotation.

GSEA was performed on mRNA expression datasets of HeLa cells with stable SKA3 overexpression and cells expressing the control plasmid using the C2 curated gene sets and C6 oncogenic signature gene sets (GSEA, Broad Institute) with the addition of a cell cycle signature. Gene signatures were considered enriched at FDR q-values < 0.05 and Family Wise Error Rate (FWER) p-values < 0.05.

### Western blotting

Cells were lysed in RIPA buffer, and the lysates were sonicated and pelleted by centrifugation. Protein concentrations were measured using a BCA protein assay kit (Thermo Fisher Scientific, Waltham, MA, USA). The lysates were mixed with 5× loading buffer, boiled for 15 min, and equally loaded on 10% SDS polyacrylamide gels. After electrophoresis, the proteins were transferred to PVDF membranes (Millipore, Billerica, MA, USA) at a constant current of 350 mA for 100 min. The PVDF membranes were blocked with 5% BSA in 1× TBST for 1 h and probed overnight at 4 °C with respective primary antibodies at dilutions suggested by the manufacturers. The following antibodies were used: anti-GAPDH (1:2000; Epitomics); anti-SKA3 (1:2000; Abcam); anti-FLAG tag (1:2000; Sigma); anti-Cdk4 (1:3000; Proteintech); anti-cyclin D1 (1:3000; Proteintech); anti-Cdk2 (1:2000; Proteintech); anti-cyclin E2 (1:2000; Proteintech) anti-p-Rb (1:2000; Proteintech); anti-E2F1 (1:3000; Abcam); anti-Akt (1:3000; Abcam); anti-p-Akt (1:3000; Abcam); anti-GSK-3β (1:2000; Sigma); anti-p21 (1:2000; Abcam); anti-p15 (1:2000; Epitomics); anti-foxo1 (1:2000; Epitomics); and anti-p-foxo1 (1:2000; Epitomics). The membranes were then incubated with a species-matched HRP-conjugated secondary antibody (1:10,000, Proteintech) for 1 h. Finally, the membranes were washed 3 times with 1× TBST (10 min each), and the blots were visualized with enhanced chemiluminescence (ECL) reagent using FUJI SUPER RX film or a CCD system (Imagestation 2000 MM, Kodak, NY, USA).

### Immunofluorescence

Cells were passaged until reaching 80% confluence and fixed with 4% paraformaldehyde for 15 min, washed 3 times with PBS (10 min each), permeabilized with 0.25% Triton X-100 for 10 min at room temperature, washed with 3 times PBS, and blocked with 5% BSA for 30 min. After washing with PBS, the cells were incubated at 4 °C overnight with an anti-E2F1 antibody (1:100, Abcam) and foxo1 antibody (1:50, Abcam). The cells were washed with PBS and incubated with a fluorescent-labeled secondary antibody (DyLight red 594, Abbkine, Amyjet) for 40 min in the dark and then later treated with DAPI for 10 min. Fluorescence was observed using a Leica inverted fluorescence microscope (Leica, Germany).

### Statistical analysis

Expression differences between normal tissues and CC tissues were calculated using a Chi squared test. Kaplan–Meier analysis was applied to determine the overall survival data. Cell proliferation and migration were analyzed using Student’s t test or one-way ANOVA. All statistical analyses were performed with SPSS 20.0 software (SPSS Inc.), and differences were considered statistically significant at p < 0.05. Data are representative of 3 independent experiments and presented as the mean ± SD.

## Results

### SKA3 expression was increased in CC patients and appeared to be a prognostic indicator of CC

**Fig. 1:**
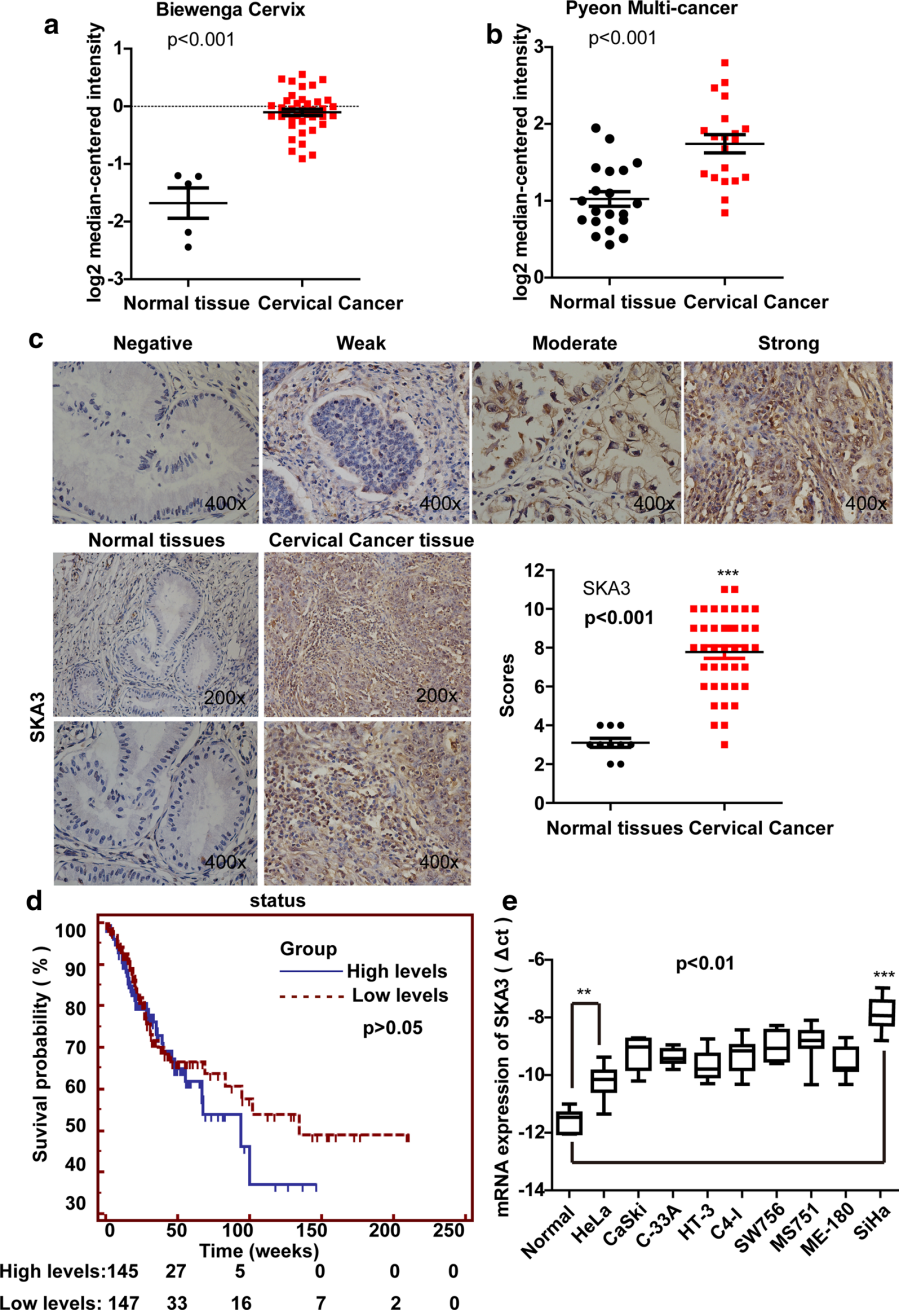
Expression of SKA3 is higher in CC tissue and appears to be correlated with overall patient survival. Data from the Oncomine database (https://www.oncomine.org/) showed that mRNA expression of SKA3 is higher in CC tissue than in normal tissue. **a** Biewenga Cervix database; **b** Pyeon Multi-cancer database. The p-value was calculated using Student’s t test (*p < 0.05, **p < 0.01). **c** Respective immunochemistry staining in a clinical tissue microarray of samples from CC patients and normal controls. **d** In a TCGA dataset, the overall survival of 292 CC patients was analyzed by Kaplan–Meier analysis. There was no significant difference between the two groups (p > 0.05, p = 0.08, patients with a high level of SKA3 vs. patients with a low level of SKA3). **e** The mRNA expression of SKA3 in different CC cell lines and normal cells; the levels of SKA3 in CC cells were higher than those in normal cervical cells (*p < 0.05, **p < 0.01)

The SKA3 gene, which is located on chromosome 13q, is associated with mitosis and cancer development. To determine the role of SKA3 in CC, we first analyzed data from the Oncomine database. We found the expression of SKA3 mRNA was higher in CC tissue than in normal tissue in both the Biewenga Cervix database (p < 0.001, Fig. 1a) and the Pyeon Multi-cancer database (p < 0.001, Fig. 1b). Next, we evaluated SKA3 expression in clinical CC patients by IHC staining of a human tissue microarray. The adjusted clinical characteristics included age, sex, pathological grade, American Joint Committee on Cancer (AJCC) stage, and TNM stage. SKA3 immunoreactivity was observed in the nucleolus and cytoplasm of both non-neoplastic epithelium and cancer cells, and SKA3 staining was obviously stronger in CC tissue than in normal tissue (score > 6, **p < 0.01, Fig. 1c), indicating that SKA3 may play an important role in CC development.

In addition, we downloaded raw survival data and SKA3 expression in CC from the TCGA Bio Portal (http://www.cbioportal.org/). The cut-off value was determined based on the median expression of individual genes. The raw survival rate of the 292 patients did not show significant differences (p = 0.08, Fig. 1d), mainly because of the small sample size and high rate of death among CC patients. According to the data, SKA3 expression may be associated with poor prognosis in CC. In addition, we examined the level of SKA3 mRNA expression in different CC cell lines and normal cervical cells, and the results suggested that SKA3 mRNA expression is higher in CC cells than in normal cells (**p < 0.01, Fig. 1e).

### SKA3 overexpression promoted cell proliferation, clone formation and migration in CC cells

**Fig. 2:**
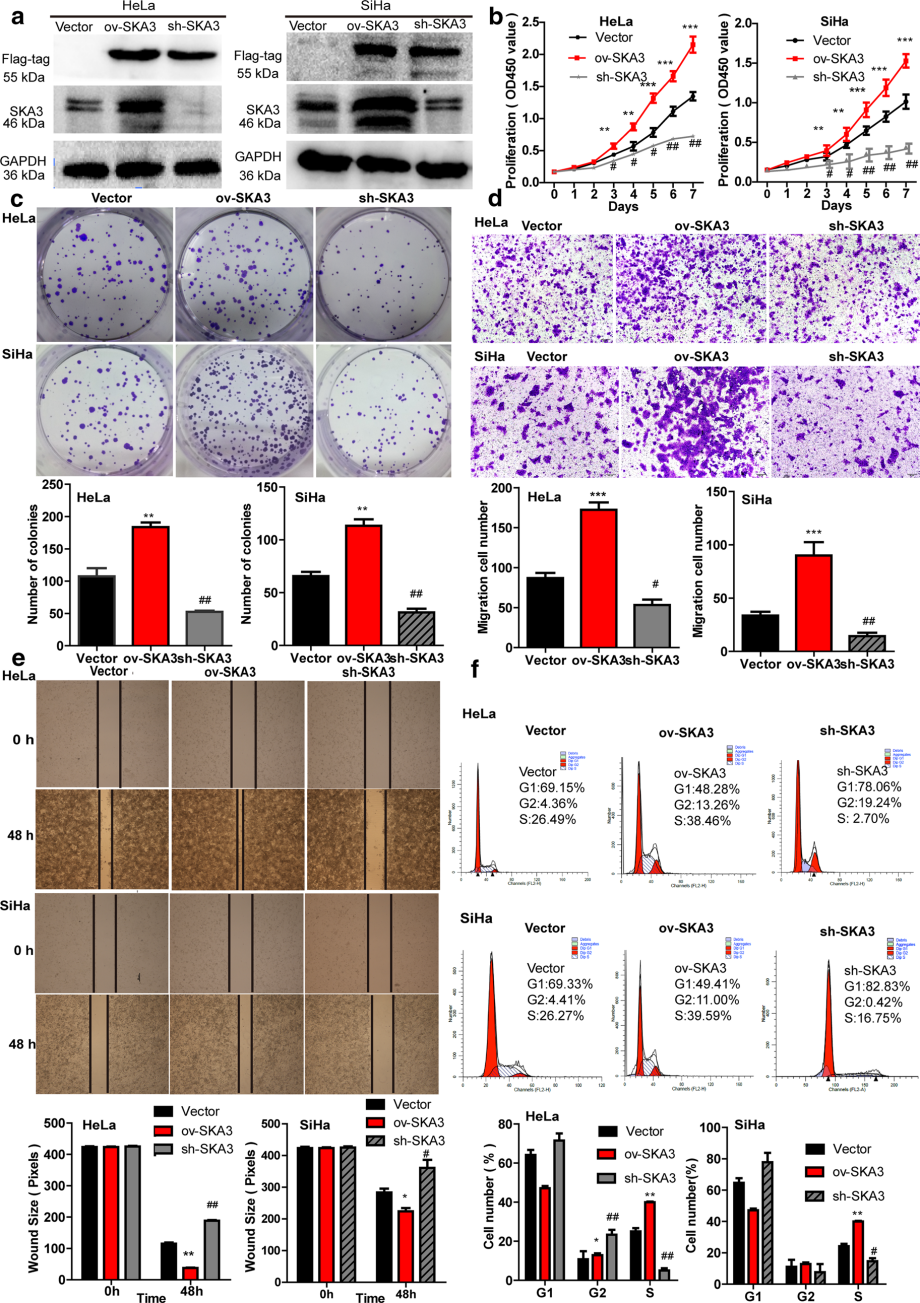
Overexpression of SKA3 promotes HeLa and SiHa cell proliferation, clone formation and migration. **a** HeLa and SiHa cells with stable SKA3 overexpression, SKA3 knockdown and control plasmid expression were generated and confirmed by western blotting. **b** Proliferation of HeLa and SiHa cells with stable SKA3 overexpression, SKA3 knockdown and control plasmid expression was assessed using a CCK-8 assay. **c** Clone formation assay in HeLa and SiHa cells with stable SKA3 overexpression, SKA3 knockdown and control plasmid expression. The migration of HeLa and SiHa cells was examined by **d** a Transwell assay and **e** a wound-healing assay. **f** Representative cell cycle data were measured by flow cytometry in HeLa and SiHa cells with stable SKA3 overexpression, SKA3 knockdown and control plasmid. All data (mean ± SD; n = 3) were analyzed by Student’s t test (*p < 0.05, **p < 0.01, over-SKA3 vs. control vector. ^#^p < 0.05, ^##^p < 0.01, SKA3 knockdown vs. control vector)

To investigate the biological function of SKA3 on CC in vitro, stable CC cell lines (HeLa and SiHa) transfected with SKA3 overexpression, knockdown and control plasmids were established and confirmed by fluorescence microscopy and western blotting. Fluorescence microscopy showed that exogenous green fluorescent protein (GFP) was well expressed in HeLa and SiHa cells. Western blotting indicated that the level of SKA3 was significantly increased in HeLa and SiHa cells with SKA3 overexpression, however the opposite result was observed in cells with SKA3 knockdown, in which the FLAG tag was measured (vs. control vector, Fig. 2a). Next, we performed a CCK-8 assay to determine the effect of SKA3 on cell proliferation ability, and the results showed that SKA3 overexpression significantly promoted HeLa and SiHa cell proliferation from day 3 to day 7, but that SKA3 knockdown significantly suppressed HeLa and SiHa cell proliferation (vs. control vector, **p < 0.01, ^##^p < 0.01, Fig. 2b). Furthermore, a clone formation assay indicated that SKA3 overexpression significantly increased the number of HeLa and SiHa cell clones (**p < 0.01, ^##^p < 0.01, Fig. 2c).

In addition, we determined whether SKA3 can influence the migration of CC cells. A Transwell assay indicated that SKA3 overexpression promoted migration in HeLa and SiHa cells, whereas SKA3 knockdown suppressed the migration in both cell types (**p < 0.01, ^##^p < 0.01, Fig. 2d). Similar results were obtained in a wound-healing assay. Whereby the wound distance was narrower in HeLa and SiHa cells with stable SKA3 overexpression than in control cells after 48 h (**p < 0.01, ^##^p < 0.01, Fig. 2e). To further understand how SKA3 affects cell growth in CC, we performed a cell cycle analysis by flow cytometry. With stable SKA3 overexpression in HeLa and SiHa cells, the percentage of cells in G1 phase and G2 phase decreased, whereas that of cells in S phase increased (vs. control vector, *p < 0.05, **p < 0.01, Fig. 2f), indicating that SKA3 overexpression promoted cell proliferation in HeLa and SiHa cells by enhancing cell cycle progression. However, the effect of SKA3 overexpression and knockdown on cell apoptosis in HeLa and SiHa cells was not significant (vs. control vector, Additional file 1: Fig. S1). In general, our results demonstrate that SKA3 overexpression promotes cell proliferation, clone formation and migration in CC cells in vitro.

### SKA3 overexpression accelerated CC tumor growth in vivo

**Fig. 3:**
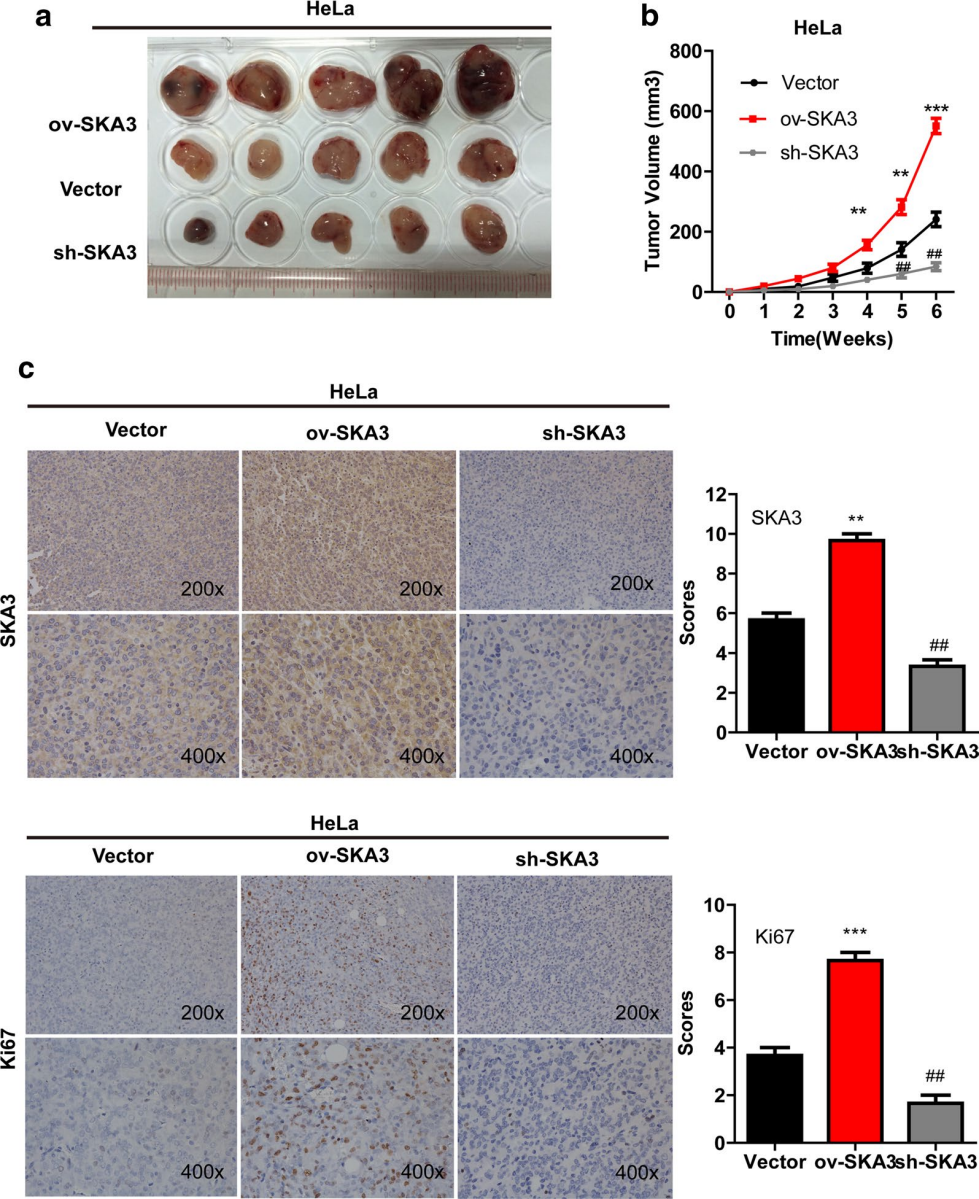
Overexpression of SKA3 accelerates CC tumor growth in vivo. **a** Representative picture of xenograft tumors formed. **b** Growth curves of xenograft tumors derived from HeLa cells with stable SKA3 overexpression, SKA3 knockdown and control plasmid expression. **c** Representative images of IHC staining for SKA3 and Ki67 in tumor sections (magnifications ×200). Data (mean ± SD; n = 6) were analyzed by Student’s t test (*p < 0.05, **p < 0.01, over-SKA3 vs. control vector. ^#^p < 0.05, ^##^p < 0.01, SKA3 knockdown vs. control vector)

To determine the effect of SKA3 on CC tumor growth in vivo, xenograft mouse models were established by injecting HeLa cells with stable SKA3 overexpression into the left dorsal flank, cells with stable SKA3 knockdown into the right dorsal flank, and cells stably expressing the control plasmid into the middle dorsal flank of different animals. Tumor size was measured continually from 1 to 6 weeks. Our results showed that SKA3 overexpression significantly increased xenograft tumor growth (Fig. 3a). The tumor volume was significantly increased in mice injected with HeLa cells stably overexpressing SKA3 from week 2 to week 6, whereas SKA3 knockdown inhibited tumor growth (vs. control vector, *p < 0.05, **p < 0.01, Fig. 3b). Furthermore, IHC staining was performed to evaluate the expression of Ki67, which is the most common indicator of cell proliferation. Ki67 and SKA3 levels were significantly increased in tumors with SKA3 overexpression (vs. control vector, *p < 0.05, **p < 0.01, Fig. 3c). Thus, we demonstrated SKA3 accelerates CC tumor growth in vivo.

### SKA3 overexpression promoted cell cycle progression by activating the PI3K–Akt pathway in CC

**Fig. 4:**
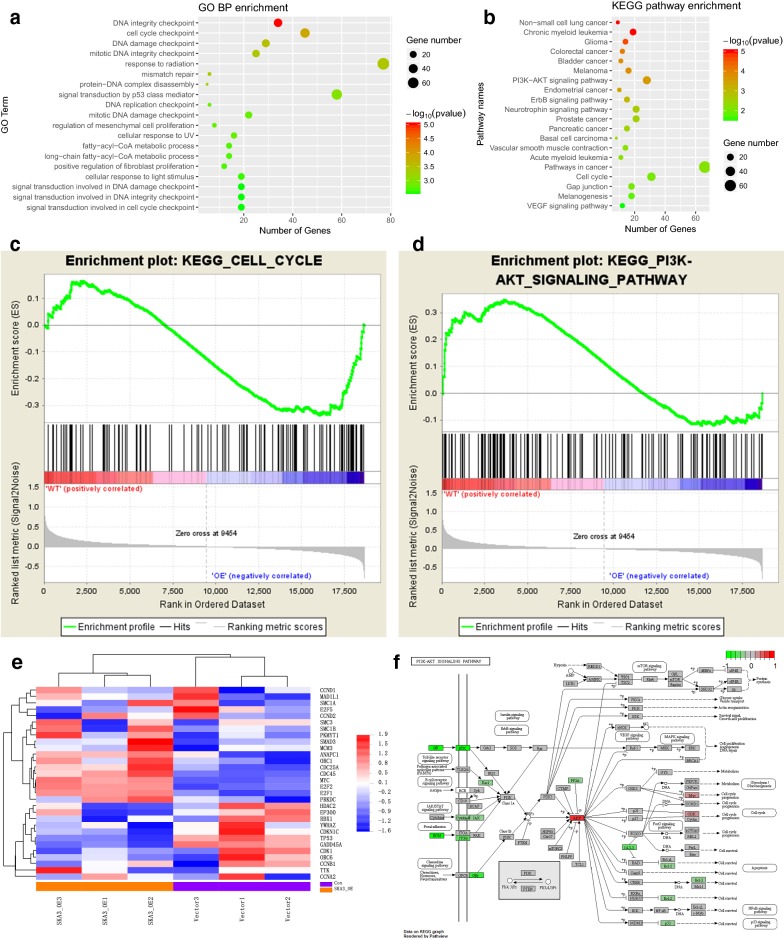
Overexpression of SKA3 facilitates the G1-S cell cycle transition and regulates the PI3K–Akt pathway in CC. **a** RNA-sequencing (RNA-Seq) analysis of genes in HeLa cells with stable SKA3 overexpression indicated involvement of the cell cycle. **b** KEGG pathway enrichment analysis (representative pathways) of genes in HeLa cells with stable SKA3 overexpression, including the PI3K–Akt pathway and cell cycle-associated genes. **c** Gene set enrichment analysis (GSEA) of HeLa cells with stable SKA3 overexpression for gene signatures of cell cycle- and **d** PI3K–Akt pathway-regulated genes. **e** Heat map of expression in the cell cycle gene set. **f** KEGG analysis of genes involved in the PI3K–Akt pathway and cell cycle

Because our results indicate that SKA3 might promote cell proliferation and cell cycle progression by favoring the G1-S transition, we next explored the molecular mechanisms of SKA3 in cell cycle progression. We evaluated gene expression using RNA-Seq analysis of mRNA isolated from HeLa cells with stable SKA3 overexpression and control vector expression. In GO enrichment, the term “cell cycle checkpoint” passed the filtering criteria (p = 0.0096, Fig. 4a). According to pathway enrichment based on the KEGG database, 19 pathways passed the filtering criteria, including the “PI3K/Akt signaling pathway” (p = 0.00027) and “cell cycle” (p = 0.0096, **p < 0.01, a representative pathway is shown in Fig. 4b). In addition, we performed GSEA for KEGG enrichment from MsigDB, and “PI3K/Akt signaling pathway” and “cell cycle” also passed the filtering criteria (**p < 0.01, Fig. 4c, d). The heat map shown in Fig. 4e indicates that the expression profiles of cell cycle-associated genes were substantially altered in HeLa cells overexpressing SKA3 (**p < 0.01). In an effort to determine the key downstream target of SKA3, we found two genes (Akt and CDK) involved in the PI3K/Akt pathway to be increased in HeLa cells overexpressing SKA3 (**p < 0.01, Fig. 4f).

**Fig. 5:**
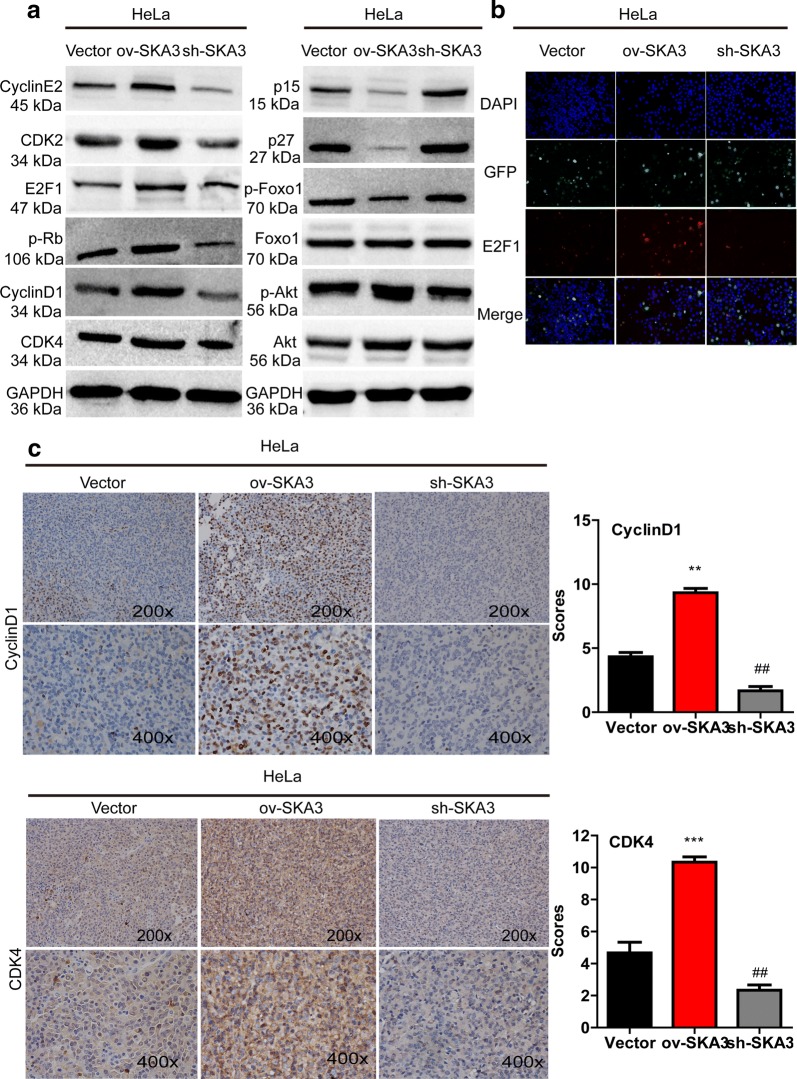
Overexpression of SKA3 promotes cell cycle progression by activating the PI3K–Akt pathway. **a** Representative western blot results of proteins related to the PI3K–Akt pathway and the cell cycle in HeLa cells with stable SKA3 overexpression, SKA3 knockdown and control plasmid expression. **b** Activity of E2F1 in HeLa cells with stable SKA3 overexpression, SKA3 knockdown and control plasmid expression by immunofluorescence. **c** Representative images of IHC staining for proteins related to the cell cycle (cyclin D1 and CDK4) in tumor sections with SKA3 overexpression, SKA3 knockdown and control plasmid expression (*p < 0.05, **p < 0.01, over-SKA3 vs. control vector. ^#^p < 0.05, ^##^p < 0.01, SKA3 knockdown vs. control vector)

Furthermore, we detected the expression of G1-S checkpoint proteins by western blotting. Levels of cyclin D1, cyclin E2, CDK4, p-Rb, E2F1 and p-Akt were higher in HeLa cells overexpressing SKA3, but levels of p15 and p27 were lower (vs. control vector, Fig. 5a). Immunofluorescence results further showed that SKA3 overexpression increased luciferase activity driven by the E2F motif (Fig. 5b). Because expression of cell cycle-related genes was altered in vitro, we also performed IHC staining to examine regulation in vivo. Expression of CDK4 and cyclin D1 was increased in tumor sections with SKA3 overexpression (vs. control vector, **p < 0.01, Fig. 5c) but decreased in tumor sections with SKA3 knockdown. Taken together, our results show that SKA3 overexpression promoted cell cycle progression by activating the PI3K/Akt pathway.

### Blocking the PI3K–Akt pathway restored SKA3-induced cell proliferation and migration

**Fig. 6:**
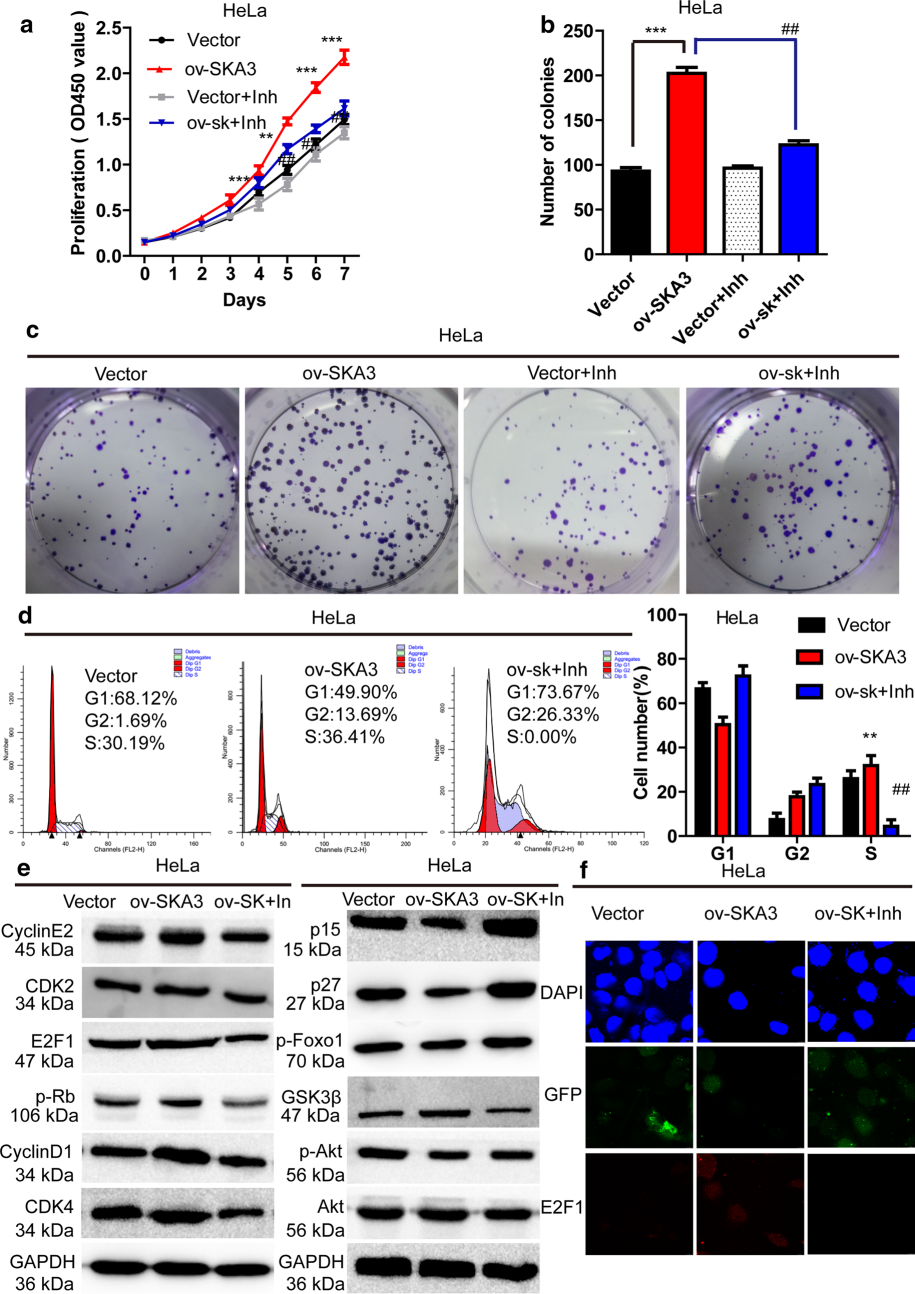
An inhibitor of Akt blocked the cell proliferation abilities induced by SKA3 overexpression in HeLa cells. **a** The proliferation curves for HeLa cells with control vector expression, SKA3 overexpression, vector expression + inhibitor treatment and SKA3 overexpression + inhibitor treatment according to a CCK8 assay. **b** Representative images of clone formation in HeLa cells with control vector expression, SKA3 overexpression, vector expression + inhibitor treatment and SKA3 overexpression + inhibitor treatment. **c** Clone formation assay in HeLa cells with control vector expression, SKA3 overexpression, vector expression + inhibitor treatment and SKA3 overexpression + inhibitor treatment. **d** Representative cell cycle data were measured by flow cytometry in HeLa cells with control vector expression, SKA3 overexpression and SKA3 overexpression + inhibitor treatment. **e** Representative western blot analyses of proteins related to the PI3K–Akt pathway and the cell cycle in HeLa cells with control vector expression, SKA3 overexpression and SKA3 overexpression + inhibitor treatment. **f** Activity of E2F1 in HeLa cells with control vector expression, SKA3 overexpression and SKA3 overexpression + inhibitor treatment

To validate our predictions, we used the Akt inhibitor GSK690693 to block Akt activity in HeLa cells overexpressing SKA3. Cells were divided into 4 groups: stable vector expression, stable SKA3 overexpression, stable vector expression + Akt inhibitor and stable SKA3 overexpression + Akt inhibitor. CCK-8 assays showed that proliferation in HeLa cells overexpressing SKA3 was significantly inhibited by GSK690693 treatment from day 2 to day 7 (vs. over-SKA3, p < 0.001, Fig. 6a). In addition, clone formation assays indicated that SKA3 overexpression significantly increased the number of HeLa colonies, and this effect was reversed by GSK690693 (vs. SKA3 overexpression, ^#^p < 0.05, ^##^p < 0.01, Fig. 6b, c). Besides, cell cycle analysis revealed that SKA3 overexpression significantly promoted the process of S phase, the effect was reversed by GSK690693 (vs. SKA3 overexpression, ^#^p < 0.05, ^##^p < 0.01, Fig. 6d). We also examined proteins downstream of Akt and related to the cell cycle. Western blotting showed that levels of p-Akt, cyclin D1, CDK4, CDK2, p-Rb, and E2F1 were increased in HeLa cells overexpressing SKA3, and this induction was blocked by GSK690693 (Fig. 6e). Furthermore, E2F1 activity was increased in HeLa cells overexpressing SKA3, an effect that was reversed by GSK690693 (Fig. 6f). Consequently, our results show that SKA3 overexpression promotes cell HeLa cell proliferation and migration by enhancing cell cycle progression and activating the PI3K/Akt pathway.

## Discussion

Despite the use of the HPV vaccine and early detection methods, identifying new molecular mechanisms and novel target therapies is extremely important for CC treatment. Using an Oncomine dataset, we found that mRNA expression of SKA3 was higher in CC tissue than in normal tissue, thus, SKA3 may play a very important role in CC progression. In our study, the expression of SKA3 in CC patient tissue was higher than that in normal tissue, and similar results were found between CC cell lines and normal cells. In addition, survival data from a TCGA dataset indicated that SKA3 overexpression appears to be associated with poor survival in CC patients. Next, we aimed to investigate the function of SKA3 in CC, and the results suggested that SKA3 overexpression promotes HeLa cell proliferation, clone formation, migration and cell cycle progression and accelerates tumor growth in vivo. Mechanistically, we provided evidence that SKA3 promotes cell cycle progression via the PI3K–Akt pathway. Taken together, our results indicated that SKA3 may serve as a useful diagnostic and therapeutic target for CC and may be used to predict the prognosis of CC patients.

SKA3 localizes to the spindle and KT throughout mitosis and along spindle microtubules [30, 31] and has multiple functions in promoting mitotic progression [32], by phosphorylating Cdk1 in mitosis, binding to Ndc80C and recruiting the Ska complex to kinetochores  [33]. Some studies have shown that SKA3 participates in cancer development. SKA3 depletion effectively blocks mitotic progression in asynchronously growing HeLa S3 cells [34]. An early study showed that SKA3 correlates with the progression of colorectal cancer, leading to higher chromosome instability (CIN) in tumors. Knockdown of SKA3 in CRC cells dramatically reduces cell growth rates, induces G2/M arrest and decreases migration and invasion [35]. In contrast, another study demonstrated that SKA3 overexpression significantly decreases migration in PC-3 [15]. In our study, as expected, the results showed that SKA3 overexpression promotes the cell proliferation, clone formation and migration in HeLa cells, which is partially consistent with the results found in CRC. Interestingly, we found no significant differences in cell apoptosis between HeLa cells with SKA3 overexpression/knockdown and cells expressing a control vector (p > 0.05). Nonetheless, our cell cycle data analysis showed that SKA3 promotes cell cycle progression mainly by facilitating the G1-S transition.

In addition, we constructed a xenograft tumor model by injecting HeLa cells overexpressing SKA3 or the control plasmid and found that expression of Ki67 was higher in tumors generated by HeLa cells overexpressing SKA3. Ki67 is a common proliferation marker, and the presence of tandem Ki67 in CC indicates a poor prognosis [36]. Our results suggest that SKA3 overexpression accelerates tumor growth and may serve as a predictor of poor prognosis in CC. To our knowledge, this is the first report that SKA3 can promote tumor growth of CC in vivo. We have thus shown that SKA3 promotes cell proliferation of CC both in vitro and in vivo.

Furthermore, RNA-Seq was used to investigate the mechanism of SKA3 as a carcinogenic gene in CC, and the results revealed alterations in genes related to the cell cycle and the PI3K–Akt pathway were altered. The cell cycle is a complex and highly orchestrated process that involves numerous regulatory proteins [37]. Previous studies of the cell cycle in CC have mostly concentrated on G2/M phase [38, 39], whereas only a few studies have focused on the G1/S transition in CC [40]. Although a previous study showed that knockdown of SKA3 induced G2/M arrest in CRC cells, our results indicated that SKA3 promotes cell cycle progression in HeLa cells by enhancing the G1-S transition. Key cell cycle regulators that govern the progression of cells from G1 to S phase include Rb-E2F1, cyclin-dependent kinase (cdk) complexes and cdk inhibitors [41–43]. Additionally, western blotting results revealed that SKA3 overexpression up-regulated the level of cell cycle-related proteins such as cyclin D1, CDK4, CDK2, cyclin E2, and p-Rb and promoted the activity of E2F1. Mitogenic stimulation during G1 phase leads to sequential activation of cdk4/6-cyclin D and cdk2–cyclin E complexes, which hyperphosphorylate Rb and thereby cause the release of active E2F1 [44], which controls expression of downstream genes essential for transition from G1 to S [45]. These findings support our western blot results.

PI3K/Akt pathway is associated with characteristics of carcinogenesis and is frequently activated in numerous human cancer types, such as in non-small cell lung cancer [46], colorectal cancer [47], breast cancer [48] and prostate cancer [49]. In addition, a recent study showed that women with CC commonly display PI3K pathway alterations, indicating its potential value in the treatment of advanced and metastatic disease [50]. In the present study, western blotting results showed changes in the expression levels of genes downstream of the PI3K/Akt pathway. SKA3 overexpression up-regulated the level of p-Akt, which modulates cell cycle proteins such as cyclin D1, CDK4, CDK2, cyclin E2 and E2F1 while inhibiting the activity of foxo1, p15 and p27. In cancer cells, increased activation of p-Akt can promote cell growth by regulating cell cycle regulators, such as p21 and p27 [51], which supports our results. Furthermore, we blocked the PI3K/Akt signaling using an Akt inhibitor (GSK690693) in HeLa cells with stably overexpressing SKA3 or the control plasmid, which reversed cell proliferation and clone formation capacities induced by SKA3 overexpression. Additionally, western blotting demonstrated that the levels of p-Akt, cyclin E, CDK2, CDK4 and cyclin D1 were down-regulated, but those of foxo1, p15 and p27 up-regulated by the Akt inhibitor in HeLa cells. Thus, we propose that SKA3 overexpression promotes cervical cancer cell proliferation and migration by regulating the cell cycle and the PI3K/Akt pathway, which might suggest a new strategy for finding novel targetable pathways in CC.

## Conclusion

In short, we suggest that SKA3 promotes cell proliferation and migration by promoting cell cycle progression and PI3K/Akt signaling pathway in CC. It may be a promising therapeutic candidate and prognostic indicator in CC.

## Additional file

**Additional file 1: Fig. S1.** Overexpression of SKA3 does not affect apoptosis in HeLa cells. (C) Representative apoptosis data, as measured by flow cytometry in (A) HeLa cells and (B) SiHa cells with stable SKA3 overexpression, SKA3 knockdown and control plasmid expression. All data (mean ± SD; n = 3) were analyzed by Student’s t test (*p < 0.05, **p < 0.01, over-SKA3 vs. control vector. ^#^p < 0.05, ^##^p < 0.01, SKA3 knockdown vs. control vector.

## Abbreviations

SKA3: spindle and kinetochore-associated complex subunit 3
CC: cervical cancer
IHC: immunohistochemistry
CDK: cyclin-dependent protein kinase
Rb: retinoblastoma
