# Supplementary figures and images for: SKA3 promotes cell proliferation and migration in cervical cancer by activating the PI3K/Akt signaling pathway
Source: Cancer Cell Int. 2018 Nov 14;18:183. doi: 10.1186/s12935-018-0670-4 (PMC6236911; doi:10.1186/s12935-018-0670-4)

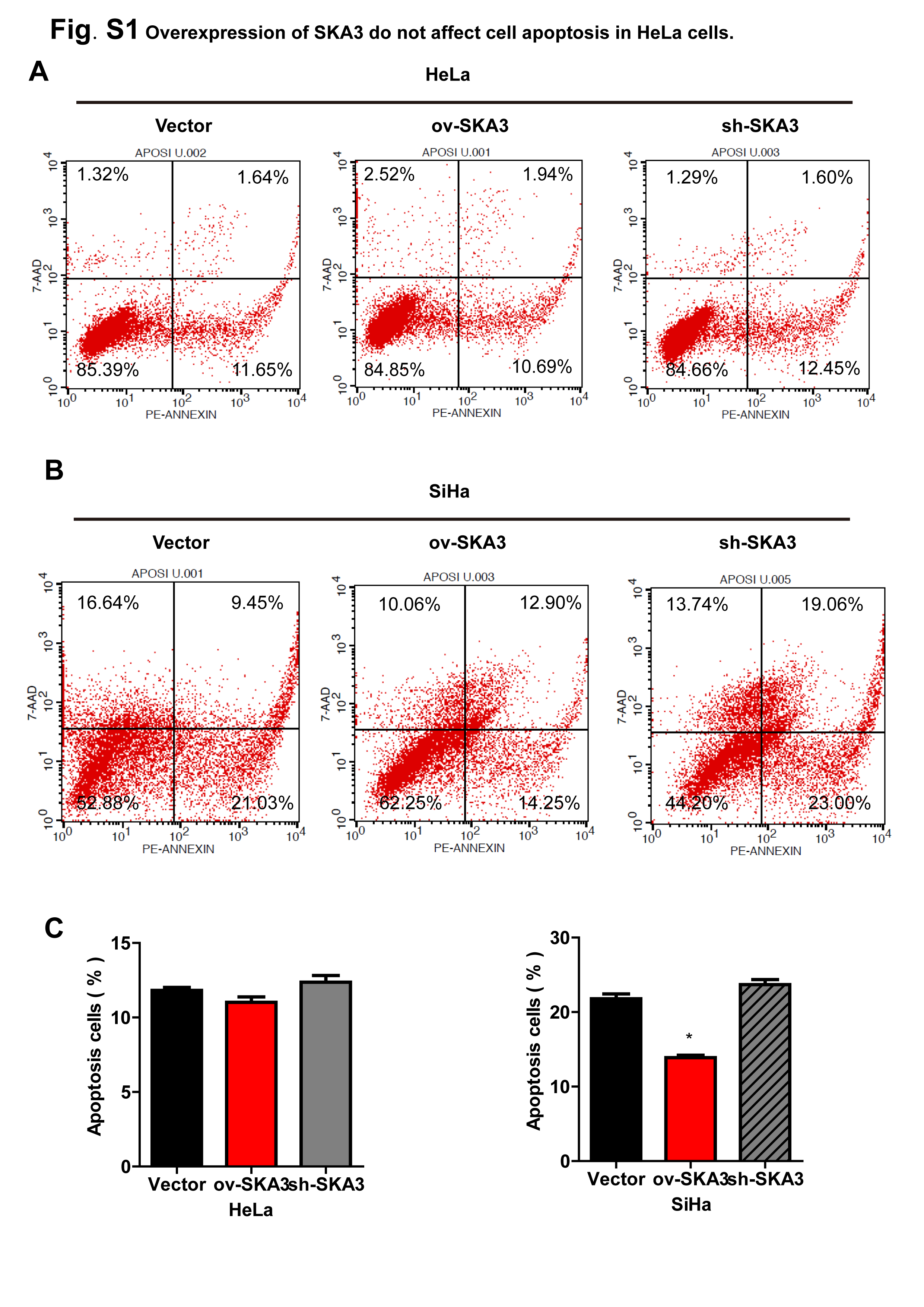

Supplement: Supplementary file 1 — Additional file 1: Fig. S1. Overexpression of SKA3 does not affect apoptosis in HeLa cells. (C) Representative apoptosis data, as measured by flow cytometry in (A) HeLa cells and (B) SiHa cells with stable SKA3 overexpression, SKA3 knockdown and control plasmid expression. All data (mean ± SD; n = 3) were analyzed by Student’s t test (*p < 0.05, **p < 0.01, over-SKA3 vs. control vector. #p < 0.05, ##p < 0.01, SKA3 knockdown vs. control vector. [file 12935_2018_670_MOESM1_ESM.tif]
